# Enhanced Refinement of Al-Zn-Mg-Cu-Zr Alloy via Internal Cooling with Annular Electromagnetic Stirring above the Liquidus Temperature

**DOI:** 10.3390/ma12142337

**Published:** 2019-07-23

**Authors:** Tianyang Guan, Zhifeng Zhang, Yuelong Bai, Bao Li, Ping Wang

**Affiliations:** 1 General Research Institute for Non-Ferrous Metals, No.2, Xinjiekouwai Street, Xicheng District, Beijing 100088, China; 2 Key Laboratory of Electromagnetic Processing of Materials, Ministry of Education, Northeastern University, No. 3-11, Wenhua Road, Shenyang 110004, China

**Keywords:** Al-Zn-Mg-Cu-Zr alloy, grain refinement, internal cooling, electromagnetic stirring, above liquidus

## Abstract

There are two critical stages of grain refinement during solidification: above and below the liquidus temperature. The key to improve the refinement potential is ensuring the nucleation sites precipitate in large quantities and dispersed in the melt above liquidus. In this work, internal cooling with annular electromagnetic stirring was applied to an Al-Zn-Mg-Cu-Zr alloy at a temperature above liquidus. A systematic experimental study on the grain refining potential was performed by combining different melt treatments and pouring temperatures. The results indicate that internal cooling with annular electromagnetic stirring (IC-AEMS) had a significantly superior grain refining potency for the alloy compared to traditional electromagnetic stirring (EMS). In addition, homogeneous and refined grains were achieved at high pouring temperatures with IC-AEMS. The possible mechanisms for the enhanced grain refinement above the liquidus temperature are explained as the stable chilling layer around the cooling rod in IC-AEMS providing undercooling for the precipitation of Al_3_Zr nucleant particles and the high cooling rate restraining the growth rate of these particles. At the same time, forced convection promotes a more homogeneous distribution of nucleant particles.

## 1. Introduction

Grain refinement is a crucial step in material processing, and has been the subject of much study [1,2]. Chemical inoculation is utilized extensively in the grain refining of aluminum and magnesium alloys. Upon the addition of a proper grain refiner, which act as potent nucleation sites, are released and dispersed into a melt [3,4]. Ramirez et al. demonstrated that the refining effect depended primarily on the selection of a nucleating catalyst [5]. Though they are easy to apply, the refining potency will deteriorate after a prolonged holding time because of the agglomeration of particles. Greer reported that less than 1% of particles function in nucleating grains, and the vast majority of particles in refiners are very inefficient [6]. Additionally, Quested and Greer found that the size, morphology, and quantity of the particles in the refiner were important factors that determine grain refining [7]. In the solidification process, it is difficult to disperse nuclei and solute uniformly relying only on the natural convection caused by heat diffusion or composition differences in the molten alloy. Hence, an alternative to address the limitations of chemical refining is to introduce forced convection by physical means. Fan et al. improved microstructural and compositional uniformity for both Mg and Al alloys by using twin-screw mechanical stirring [8,9]. Guan et al. proposed a vibrating wavelike sloping plate process to improve heterogeneous nucleation by providing strong undercooling [10]. Robles Hernández and Sokolowski showed that electromagnetic stirring and vibration melt treatments are effective in both the liquid and semi-solid states [11]. Eskin et al. summarized the recent research on the evaluation of ultrasonic melt processing [12]. Most physical refining via forced convection promotes the columnar to equiaxed transition (CET) and provokes a distinct grain-refining effect.

As a representative, electromagnetic stirring (EMS) has been successfully applied to solidification processing for grain refinement [13,14]. It is well known that the Lorentz force caused by an alternating magnetic field can drive the forced convection and homogenize solute and temperature fields [15,16]. The most extensively accepted theory is that forced convection promotes the breaking of dendrite arms or the remelting of dendrite arm roots, which act as effective nucleation substrates. Meanwhile, Pilling and Hellawell considered that the forces caused by fluid flow could only be expected to cause bending, as opposed to fracture [17]. Recently, Wang et al. revealed that external field had an important influence on the grain refiners at different stages [18]. Thus, the detailed refinement mechanism at different stages is not yet clearly understood, and the main source of nucleation sites needs to be further investigated. 

In fact, there are two critical stages for grain refinement during solidification: well above the liquidus and then as the melt cools below the liquidus. It is impossible for the α-Al phase to nucleate and grow above liquidus, but the inoculant particles will precipitate and be stable enough in the melt to survive until solidification. Haghayeghi et al. investigated the refinement mechanism above the liquidus by shearing [19]. Wang et al. applied high-intensity ultrasonic to an Al-0.4 wt. % Ti alloy at temperature ranges from above to below the liquidus temperature, and found that the primary Al_3_Ti particles were refined over all temperature ranges [20]. Our previous research has shown that the formation of primary Al_3_Zr was influenced by internal cooling with annular electromagnetic stirring (IC-AEMS) melt treatment above the liquidus, and a remarkable grain refinement took place in the pure aluminum [21]. However, both the nuclei and solute play an important role in the grain refinement of aluminum alloys [22]. Therefore, much work needs to be done to investigate the effects of IC-AEMS melt treatment above the liquidus on subsequent nucleation and refinement potency for multi-component alloys. 

This study aimed to investigate the effect of IC-AEMS above the liquidus on the refining potency and efficiency of a multi-component Al-Zn-Mg-Cu-Zr alloy. The grain refinement was examined with three different melt treatments and four different pouring temperatures, and the possible mechanisms involved will be discussed.

## 2. Materials and Methods 

In the present work, an Al-Zn-Mg-Cu-Zr alloy was prepared by CP-Al (99.8% purity), copper (99.9% purity), zinc (99.9% purity), magnesium (99.9% purity), and master alloy Al-4%Zr (all compositions are in wt% unless otherwise specified). The practical compositions of the alloy, as determined through optical emission spectroscopy (FOUNDRY-MASTER Pro, Oxford Instruments, Oxford, UK) are listed in Table 1. The liquidus temperature of the alloy was calculated to be 624 °C using the JmatPro software (Sente Software Ltd, Guildford, UK).

**Figure 1:**
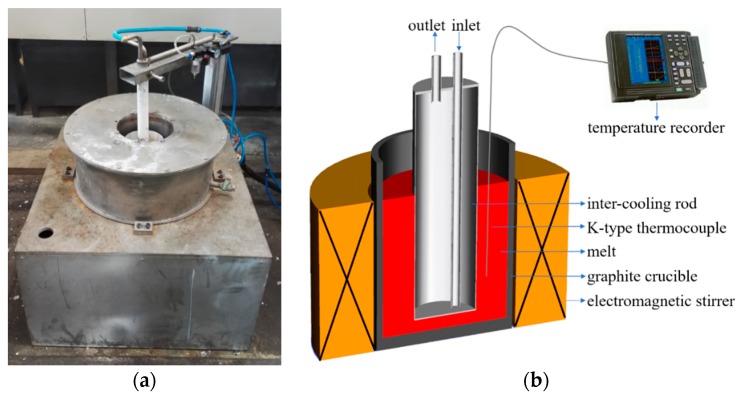
(**a**) Experimental facilities and (**b**) schematic diagram of internal cooling with annular electromagnetic stirring (IC-AEMS).

The experiments were carried out on self-made IC-AEMS equipment, and the experimental facilities and schematic illustration are shown in Figure 1. The equipment consisted of an electromagnetic stirrer, a graphite crucible, an internal cooling system, and a temperature-monitoring system. In the present experiments, the electromagnetic stirring frequency and current were imposed as 30 Hz and 60 A, respectively, and a K-type thermocouple was positioned at a distance of half the radius away from the wall to monitor the final temperature of the stirring. When the treatment temperature was adequate, the cooling rod was removed and the molten alloys were poured into a TP-1 mold (3.5 °C/s) [23]. In order to prevent a solid layer forming on the internal cooling rod, which hinders the cooling effectiveness, a boron nitride-coated cooling rod was preheated to 300 °C and was inserted into the melt immediately after the cooling air was introduced. 

The alloy was melted in a clay-bonded graphite crucible, which was placed in an electrical resistance furnace and heated to 780 °C. After degassing and skimming off the dross, the molten alloy was transferred to the melt treatment equipment. For comparison, four different pouring temperatures and three different melt treatments were adopted. The selected pouring temperatures were 670, 660, 650, and 640 °C. The stirring temperature was kept above the liquidus temperature at all times. Three sets of parallel experiments were carried out at the same temperature: without treatment (marked as Normal), with EMS, and with IC-AEMS. 

Metallographic samples for grain structure examination were prepared using standard metallographic techniques, and the detailed sampling positions can be found in [21]. The polished samples were anodized with Barker’s reagent (4% HBF_4_ in distilled water) for 50 s at 30 V. All the samples were examined under polarized light using a Zeiss optical microscope (OM, Zeiss Axiovert 200MAT) (Carl Zeiss AG, Heidenheim an der Brenz, Germany). The grain sizes were measured by the lineal intercept method [24], and the average grain density was counted using Image-pro Plus software (Media Cybernetics, Maryland, USA). The homogeneity of the chemical composition on the microstructures were characterized by scanning electron microscopy (SEM, JSM-7610F) (JEOL, Tokyo, Japan) equipped with energy dispersive X-ray spectroscopy (EDS). The Al_3_Zr crystals were investigated by a transmission electron microscope (TEM, FEI-F20) (FEI, Hillsboro, OR, USA) operating at 200 kV and selected area electron diffraction (SAED) analysis.

## 3. Results

**Figure 2:**
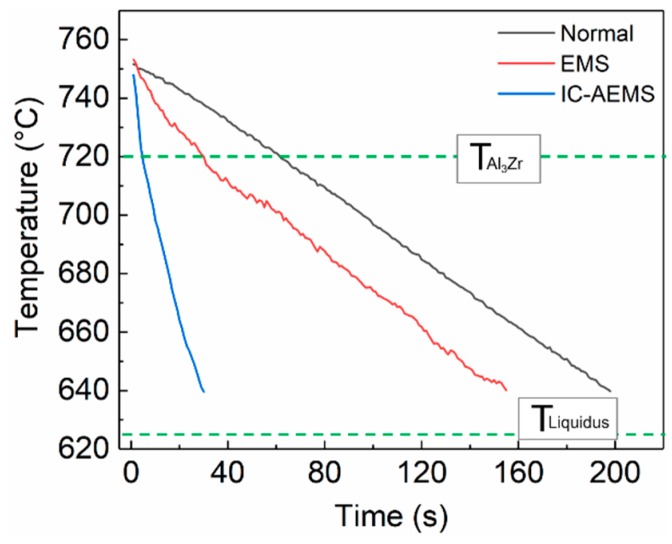
Cooling curves of Al-Zn-Mg-Cu-Zr alloy with three different treatments in liquid state. The dotted line represents the equilibrium precipitation temperature of primary Al_3_Zr and the liquidus of Al-Zn-Mg-Cu-Zr alloy.

**Figure 3:**
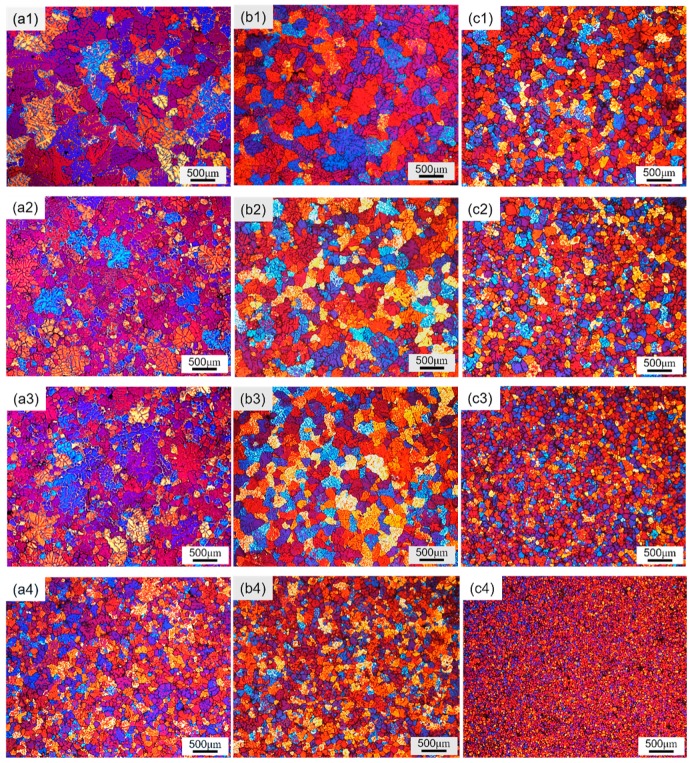
Polarized light micrographs showing the microstructural characterization of Al-Zn-Mg-Cu-Zr alloy with different melt treatments: (**a**) without treatment, (**b**) with electromagnetic stirring (EMS), (**c**) with IC-AEMS, and pouring temperature: (**1**) 670 °C, (**2**) 660 °C, (**3**) 650 °C, (**4**) 640 °C.

**Figure 4:**
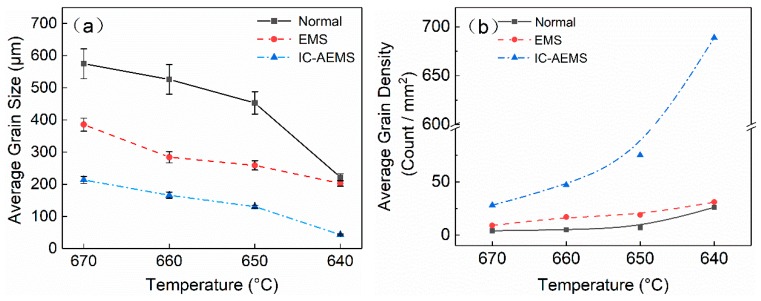
Variation of the (**a**) average grain size and (**b**) average grain density of grains with three different treatments at four selected temperatures.

Figure 2 shows the cooling curves with three different treatments in liquid state, and the cooling rates were 0.55 (Normal), 0.68 (EMS), and 3.56 °C/s (IC-AEMS). Figure 3 presents the microstructural evolution in polarized light optical micrographs under varying pouring temperatures and different melt treatment applications. In this work, three different treatment conditions (i.e., (a) without treatment, (b) with EMS, (c) with IC-AEMS) and four different pouring temperatures (i.e., (1) 670 °C, (2) 660 °C, (3) 650 °C, (4) 640 °C) were investigated. The measured average grain sizes and the average number density are presented in Table 2 and Figure 4, respectively. 

At a pouring temperature of 670 °C, typical coarse dendritic structure were observed (Figure 3 (a1)). When EMS was applied, the coarse secondary arms were improved (Figure 3 (b1)), though the coarse dendritic grains were not completely eliminated. In the case of IC-AEMS, a substantial reduction in grain size and a homogeneous equiaxed structure can be observed in Figure 3 (c1). No coarse dendritic grains were observed, and the total reduction in grain size was about 64% compared with the condition without treatment. This demonstrated that more nuclei were activated and preserved after IC-AEMS treatment. 

As the pouring temperature decreased to 660 °C and 650 °C in Figure 3 (a2,a3), the dendritic arms lapped with each other, and relatively small-sized grains were found under the normal condition. On one hand, these small grains may be the cross section of the dendrite arms; on the other hand, the nonuniform temperature and compositional field caused by the slow cooling rate and natural convection could provide conditions for the nucleation and growth restriction of some free crystals. For the samples with EMS treatment, the morphology tended to evolve from an inhomogeneous dendritic structure to rosette crystal, and the average grain size decreased to about 260 μm. This result indicates that the strong convection of molten alloys caused by EMS could effectively influence the solute distribution. As shown in Figure 3 (b2,b3), it should be noted that the grain size at a pouring temperature at 660 °C was approximately the same and no significant differences were detected in the morphology achieved by pouring at 650 °C, which means the traditional electromagnetic stirring has limitations in refining efficiency. When IC-AEMS was applied, the average grain size kept reducing as the pouring temperature decreased.

By reducing the pouring temperature to 640 °C, the refining effect on the grain was obvious across all three samples in Figure 3 (a4,b4,c4). Although the microstructure after EMS treatment tended to be more equiaxed, the refining effect was almost the same as that of the untreated sample. However, the grain size with IC-AEMS exhibited significantly higher potency than EMS, as revealed by the sharp decrease in Figure 3 (c4). As depicted in Table 2 and Figure 4, the total reduction in grain size was 80% and the number density of grains increased about 240%. It is noteworthy that, as can be seen in Figure 3 (c1,a4), the grains with IC-AEMS at 670 °C were similar to the normal grains at 640 °C. For some practical production, such as squeeze casting, the pouring temperature is much higher than the liquidus, which means it can obtain a fine and equiaxed microstructure at a high temperature after IC-AEMS treatment.

**Figure 5:**
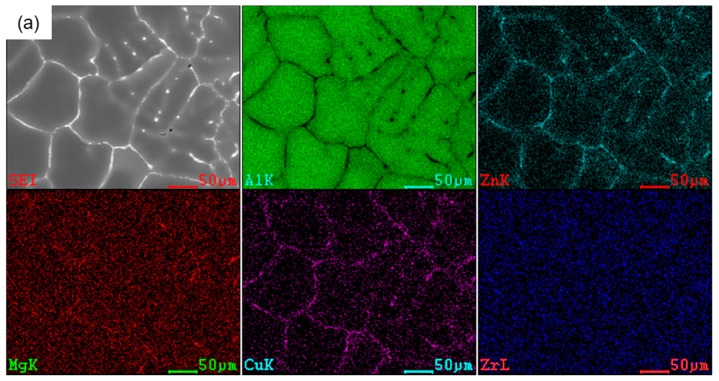

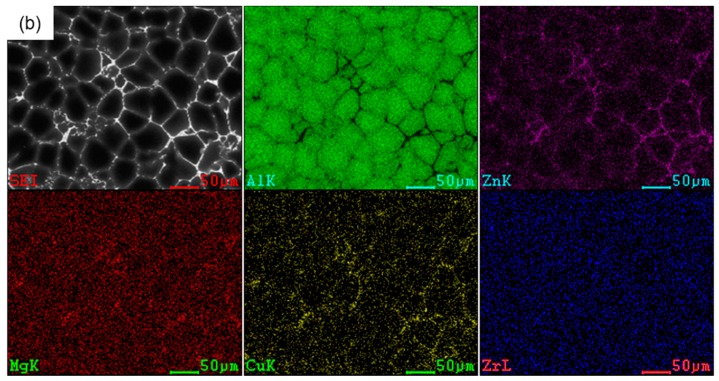
EDS mapping images analysis of the Al-Zn-Mg-Cu-Zr at 640 °C: (**a**) without treatment, (**b**) with IC-AEMS treatment.

The EDS mapping results are illustrated in Figure 5. The chemical composition was relatively homogeneous within a single grain, but the eutectic phase composed of Zn, Mg, and Cu was mainly concentrated at grain boundaries. The solute elements’ distributions were non-uniform for the molten alloy without treatment in Figure 5a. Compared with coarse grains, fine equiaxed grains showed more grain boundaries in the same area; therefore, the solute concentrations in the boundaries were improved and the solute distributions were uniform relatively uniform after being treated by IC-AEMS (Figure 5b). 

**Figure 6:**
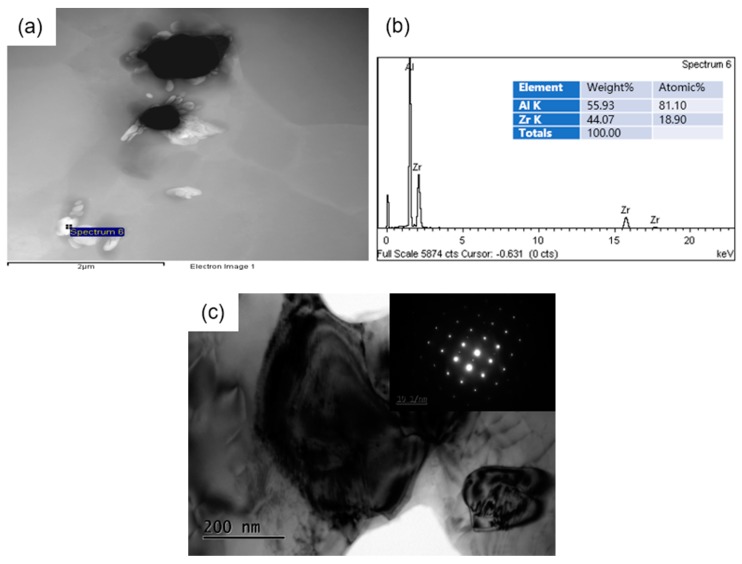
Al_3_Zr distribution in the Al-Zn-Mg-Cu-Zr alloy with IC-AEMS treatment: (**a**) TEM low magnification image; (**b**) EDS analysis corresponding to spectrum 6; (**c**) The bright-field TEM images and selected area electron diffraction (SAED) patterns taken from the particle Al3Zr and the adjacent aluminum matrix.

Note that the content of Zr was 0.2% in this work, and it is difficult to capture Al_3_Zr particles in the TP-1 samples above liquidus, especially for multi-component alloys. Figure 6 shows a TEM low magnification image and a TEM bright field image of the Al_3_Zr particle embedded in the Al-Zn-Mg-Cu-Zr alloy with IC-AEMS treatment.

**Figure 7:**
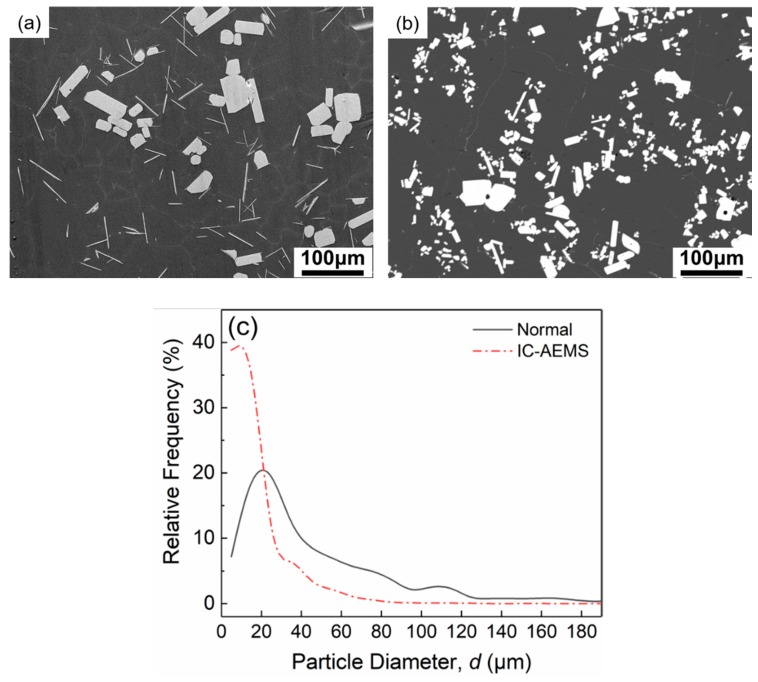
SEM micrographs of the primary Al_3_Zr particles in the deeply-etched samples: (**a**) without treatment; (**b**) with IC-AEMS. (**c**) The size distribution of Al_3_Zr particles in the Al-Zn-Mg-Cu-Zr alloy.

To make it easier to investigate the morphology of these particles, we imitated previous work and kept the molten alloy solidified at room temperature in the crucible [21], so that Al_3_Zr particles would grow and settle to the bottom during the slow cooling rate in the solidification process. Figure 7 presents the morphology and distribution of the Al_3_Zr particle diameter in Al-Zn-Mg-Cu-Zr alloy both with and without IC-AEMS treatment. The morphology was transformed from rod-like and tabular to small and blocky. After treated by IC-AEMS, a high density of small homogeneous particles were distributed in the matrix.

## 4. Discussion

The constitutional undercooling caused by solute segregation has been proven critical in determining the final grain size [25], as has the growth restriction factor *Q* used to quantify the effect of solute on the grain refinement of binary alloys. For multi-component alloys, the value of *Q* is given by the following equation [3,26,27]:

(1) $$Q=\sum_{i=1}^{N}m_{i}c_{0i}\left(k_{i}-1\right),$$

where *m_i_* is the slope of liquidus, *c_0i_* is the concentration of element in the alloy, and *k_i_* is the equilibrium partition coefficient. Very often, *m_i_* and *k_i_* values for a multi-component alloy are estimated from the binary alloy systems [28]. The data for Al-Zn-Mg-Cu-Zr alloy in this work were obtained from Ref. [29] and are listed in Table 3.

Therefore, in terms of the deficiency of constitutional undercooling in aluminum, zirconium solute should not produce effective grain refinement in multi-component alloys. Besides, the turbulence caused by forced convection would improve the distribution of solute field. However, a dramatically reduced grain size was observed, especially when IC-AEMS was applied. This implies that something else contributes to the grain refinement after IC-AEMS treatment. For the molten alloy above the liquidus, it is theoretically impossible for the α-Al phase to nucleate and grow, but the high-melting-point heterogeneous nucleant particles will precipitate. The morphology and size of these particles will influence the subsequent solidification stage as the melt cools below the liquidus temperature. According to the free growth model, the critical undercooling is related to the size of nucleant particles by the following equation [3,6]:

(2) $$\Delta T_{g}=\frac{4\sigma }{\Delta S_{V}d}$$

where Δ*T_g_* is the undercooling required, *σ* is the solid–liquid interfacial energy, Δ*S_V_* is the entropy of fusion per unit volume, and *d* is the size of the active nucleant particle. From the equation, it can be seen that the free growth undercooling is inversely related to particle size, that is, the smaller the nucleant particles, the higher the undercooling required to activate the grain initiation on the nucleant particles. Greer et al. [3] and StJohn et al. [22] also proposed that grain initiation was controlled by the linear dimension of the potent nucleant particle. In the case of IC-AEMS, the cooling rod provided an area of higher undercooling for nucleation. As shown in Figure 7, the size distribution of Al_3_Zr particles (0–200 μm) was much broader than that of the particles with IC-AEMS (0–100 μm). Additionally, more than 90% of the particles were concentrated in the range of 0–40 μm. The particle density was 3.4 × 10^3^ /mm^2^ with IC-AEMS, while under normal conditions the density was only 1.2 × 10^3^ /mm^2^.

In this work, the content of Zr (0.2%) exceeded the maximum solubility (0.11%), and Al_3_Zr formed prior to the solidification of Al [30]. The cooling rate under normal conditions was ~0.55 °C/s and the equilibrium precipitation temperature of primary Al_3_Zr was 720 °C. Under the circumstances, the primary Al_3_Zr crystals gradually grew and consumed solute Zr, which inhibited the growth of small embryos. Thus, the natural convection and lower cooling rate both led to Al_3_Zr particles with a wide range of distribution and diameter. In the case of EMS, the cooling rate increased slightly to ~0.68 °C/s, but the forced convection created by EMS affected the heat transport by dissipating the superheat and homogenizing the temperature in the melt. As a result, a larger amount of nuclei survived and distributed well within the melt, and grains became homogeneous in size during the solidification.

When IC-AEMS was introduced into the melt, the cooling rate increased to ~3.56 °C/s. The chilling layer around the cooling rod provided extreme undercooling for Al_3_Zr nucleation, which were then scoured down and dispersed into the melt by the forced convection. On one hand, the faster cooling rate made small nucleant particles less susceptible to remelting, causing them to survive in the melt; on the other hand, the faster cooling rate could restrain the growth rate of large nucleant particles. Therefore, compared to the normal condition and EMS, the improvement of inoculation by increasing the cooling rate above liquidus served as one of the main factors that contributed to the refinement of the alloy. 

Grain initiation occurred first on the biggest nucleant particles, then on the smaller particles as the undercooling was increased. For the particles without treatment, because of the broad distribution of particle sizes, the large particles were easily activated under a small undercooling, and small-sized particles were annexed via Ostwald ripening in the subsequent growth process [31]. In contrast, applying IC-AEMS resulted in a larger number of nucleant particles with smaller sizes being introduced into the melt at the solidification stage. Additionally, the forced convection promoted the compositional field more uniformly. Once the proper undercooling was reached, it allowed a high density of small and activated particles to have an opportunity to act as nucleation sites for the α-Al when the melt cooled below the liquidus temperature.

## 5. Conclusions

(1) IC-AEMS has an extra potential for the grain refinement of Al-Zn-Mg-Cu-Zr alloy when the melt is poured at the same temperatures above the liquidus. The average grain size reduced from 575 μm (without treatment) to 384 μm (EMS treatment), and then to 205 μm (IC-AEMS treatment) at 670 °C. A similar result was achieved at temperatures of 660, 650, and 640 °C.
(2) Compared with the normal condition and EMS, a fine and equiaxed microstructure was obtained with IC-AEMS at temperatures from 670 to 640 °C. Additionally, the grains with IC-AEMS at 670 °C were similar to the normal grains at 640 °C, which implies it is beneficial for practical production after IC-AEMS treatment.
(3) The possible mechanisms for refinement above the liquidus temperature were explained as the stable chilling layer around the cooling rod providing undercooling for the precipitation of small Al_3_Zr nucleant particles, and the high cooling rate by applying IC-AEMS restraining the growth rate of these Al_3_Zr particles, which act as nucleation sites for α-Al when the melt cools below the liquidus temperature.

## Figures and Tables

**Table 1:** Chemical composition of Al-Zn-Mg-Cu-Zr alloy (wt. %).

Zn	Mg	Cu	Zr	Fe	Si	Al
11.87	2.59	1.16	0.21	0.01	0.02	Bal.

**Table 2:** Average grain size and grain density measured from Al-Zn-Mg-Cu-Zr alloy solidified under different conditions and pouring temperatures.

T/°C	Normal		EMS		IC-AEMS	
	Size/μm	Density/mm^2^	Size/μm	Density/mm^2^	Size/μm	Density/mm^2^
670	575	4	386	9	214	28
660	526	5	284	16	166	46
650	452	7	259	19	131	75
640	221	26	203	31	43	689

**Table 3:** Related data of growth restriction factor *Q* in Al-Zn-Mg-Cu-Zr alloy.

Element	*k_i_*	*m_i_*	Maximum Concentration	*m*(*k* − 1)	*Q*
Zn	0.88	−3.0	94	0.4	4.7
Mg	0.51	−6.2	~3.4	3.0	7.8
Cu	0.17	−3.4	33.2	2.8	3.2
Zr	2.5	4.5	0.11	6.8	0.7
